# A clear cell sarcoma tumor of the finger with an unusual presentation misdiagnosed as a glomus tumor in an adolescent patient: A case report

**DOI:** 10.3892/ol.2026.15626

**Published:** 2026-04-28

**Authors:** Abdulrahman Alaseem, Hatim Khoja, Khalid Murrad, Sarah Alkhalife, Nouf Altwaijri, Abdullah Almawi, Abdulaziz Bin Dakhil, Abdulaziz Alhammad, Banan Alqady, Eman Najjar

**Affiliations:** 1 Department of Orthopedic Surgery, College of Medicine, King Saud University, Riyadh 12372, Saudi Arabia; 2 Department of Pathology and Laboratory Medicine, King Faisal Specialist Hospital and Research Centre, Riyadh 11211, Saudi Arabia; 3 Department of Orthopedics, Prince Sultan Military Medical City, Riyadh 11159, Saudi Arabia; 4 Department of Orthopedics, King Saud Medical City, Riyadh 12746, Saudi Arabia; 5 Department of Orthopedic Surgery, King Saud University Medical City, Riyadh 12372, Saudi Arabia; 6 Department of Radiology, King Faisal Specialist Hospital and Research Center, Riyadh 11211, Saudi Arabia; 7 College of Medicine, King Saud University, Riyadh 12372, Saudi Arabia; 8 Department of Radiology, Security Forces Hospital, Riyadh 12233, Saudi Arabia

**Keywords:** clear cell sarcoma, soft tissue sarcoma, orthopedic oncology, immunohistochemical marker, digital lesion

## Abstract

Clear cell sarcoma (CCS) is an uncommon malignancy originating from neural crest cells; this type of cancer can mimic a number of other tumors and frequently presents in the lower limbs, but is occasionally found in atypical locations. The present study describes a rare case of a 14-year-old girl presenting with a subungual swelling on the right index finger, with the initial impression of a glomus tumor, who underwent unplanned surgical resection at another institution. The histopathological analysis revealed CCS with positive margins, and the patient was therefore referred to an orthopedic oncology specialist King Khalid University Hospital (King Saud University Medical City, Riyadh, Saudi Arabia), for further management. Local and systemic staging workups confirmed localized disease; hence, local control was achieved by amputation at the base of the middle phalanx level, with negative margins achieved without chemotherapy or radiation therapy. Finger tumors may impose diagnostic challenges due to their rarity and similarity with more common benign conditions. A high index of suspicion is important to make an early, accurate diagnosis, and to avoid inappropriate management and poor outcomes. The current case is one of the few published reports of CCS arising in the finger and, to the best of our knowledge, the second pediatric case. Therefore, the present study contributes to the limited literature on CCS presenting in an unusual location such as the hand, with the aim of avoiding errors in diagnosis and management.

## Introduction

Clear cell sarcoma (CCS) is a tumor that arises from neural crest cells and accounts for ~1% of all diagnosed soft tissue sarcomas; it was first described by Enzinger in 1965 (1) and is most commonly found in tendinous sheaths and aponeuroses (1–4). CCS commonly involves the lower limbs, particularly the ankles and feet (2); however, sporadic presentations in the kidney, thorax, abdomen, head and neck have been described. Male and female patients of any age are equally affected, with a peak incidence in the third and fourth decades of life (5,6), Nevertheless, isolated cases have been reported in pediatrics and adolescents (2,7).

CCS usually presents as a small tender lump or swelling, and may involve subcutaneous tissue, the adjacent dermis and ulceration of the overlying skin (2,8). Although rare, CCS has been reported in the hand, where it can pose notable diagnostic challenges as it often resembles more benign conditions, such as glomus tumors, highlighting the importance of maintaining a high index of suspicion. The prognosis for CCS is generally poor, with reported distant metastasis rates ranging between 60 and 70%, primarily to the lungs or lymph nodes (4,9). This high risk of metastasis underscores the need for early detection and appropriate management. The present study describes an unusual case of CCS manifesting as a subungual swelling on the right index finger of a 14-year-old girl. The aim of the report is to provide a comprehensive review of the diagnostic and treatment approaches for CCS, as well as to discuss the importance of accurate clinical evaluation to avoid misdiagnosis and ensure early multidisciplinary management. While CCS is rare and similar cases have been reported (1,2), the current case represents the seventh case of CCS presenting in the finger and the second pediatric case published. Notably, to the best of our knowledge, it is the first pediatric case with a favorable prognosis and no recurrence, which adds valuable insight into the potential for long-term survival in this population of patients. These aspects may contribute to the current understanding of CCS, particularly in pediatric patients.

## Case report

**Figure 1.**
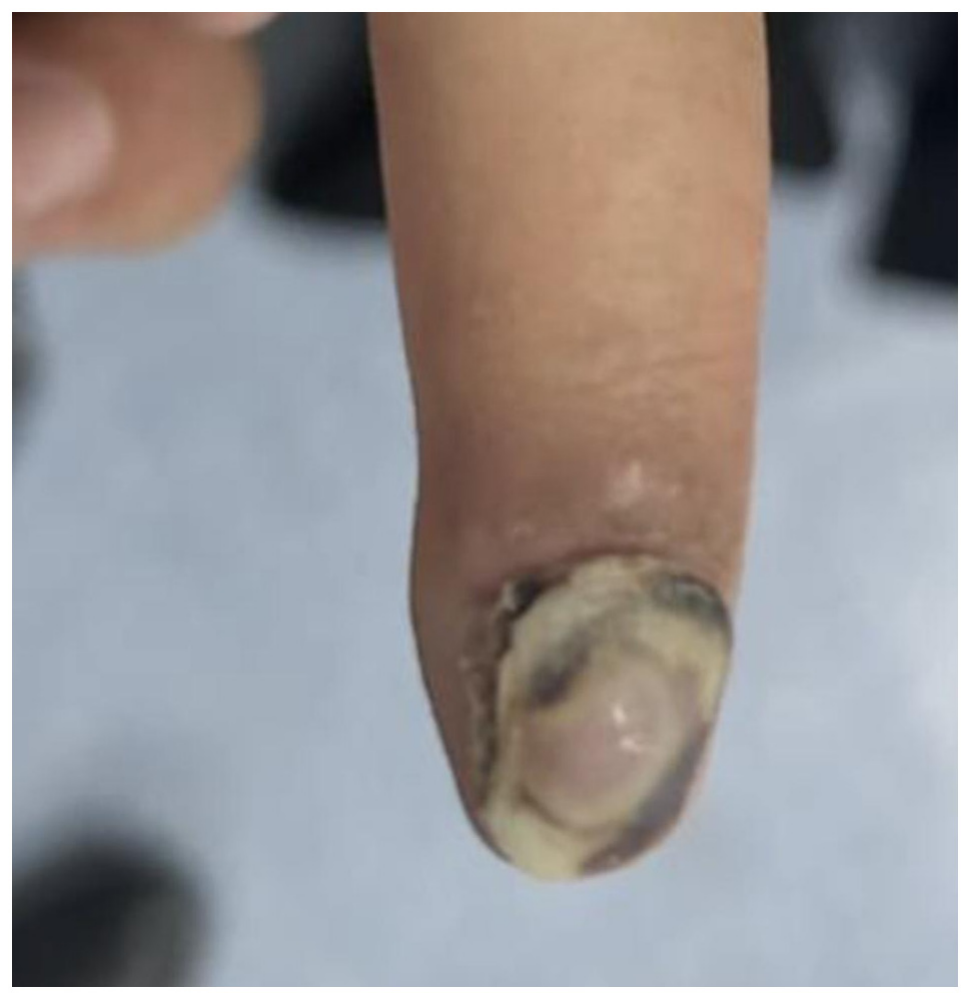
Irregular melanonychia of the nail plate associated with a central, nodular subungual mass involving the right index finger.

**Figure 2.**
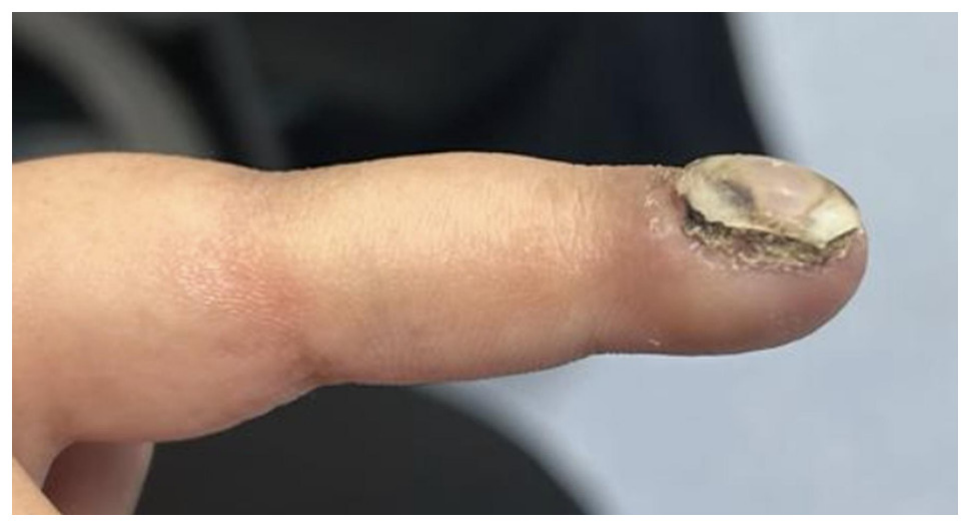
Dark discoloration, swelling, dystrophic changes of the right index finger with visible nail deformity.

The present study describes the case of a 14-year-old girl who presented to the orthopedic clinic at King Khalid University Hospital (King Saud University Medical City, Riyadh, Saudi Arabia, on January 2023 with a 4-year history of right index finger swelling, associated with severe pain, rapid progression in size over the last year, and no history of trauma or fever. The patient had no other lumps elsewhere, and denied any constitutional symptoms, or personal or family history of neoplasms. Physical examination of the right hand revealed a swelling with dark skin pigmentation of the tip of the second finger and melanonychia (brownish-black discoloration of the nail plate) associated with tenderness but no ulceration or discharge (Figs. 1 and 2). The systemic examination was unremarkable. Initial radiographic investigation with X-ray, which was performed at another institution before presenting to the aforementioned orthopedic clinic, revealed a well-circumscribed lytic lesion involving the volar aspect of the distal phalanx of the right index finger, with no evidence of pathological fracture.

**Figure 3.**
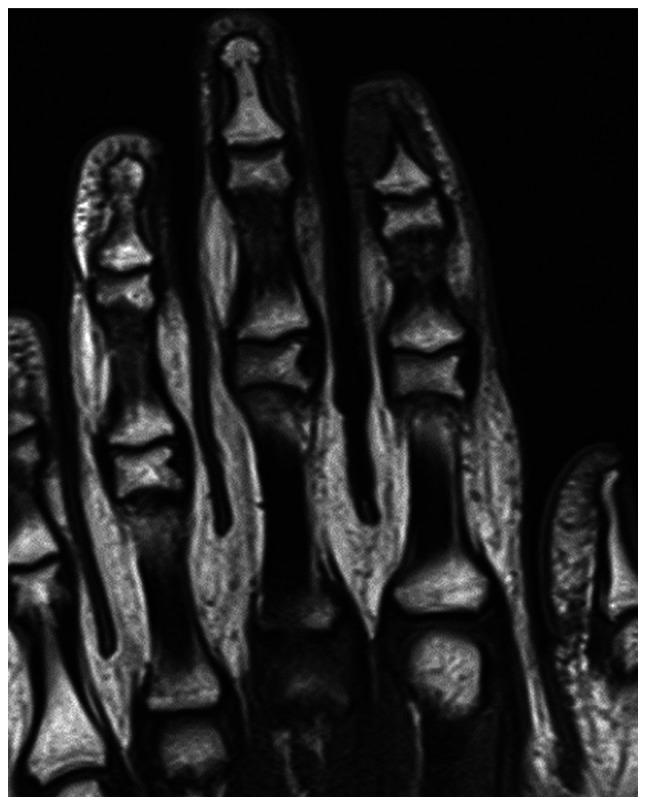
Coronal T1-weighted magnetic resonance imaging scan of the lesion in the right index finger.

**Figure 4.**
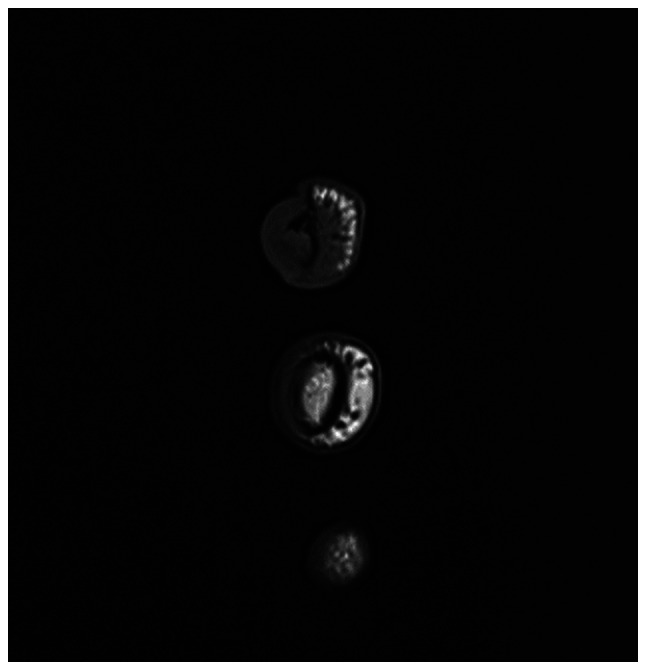
Axial T1-weighted magnetic resonance imaging scan demonstrating the lesion with low signal intensity.

**Figure 5.**
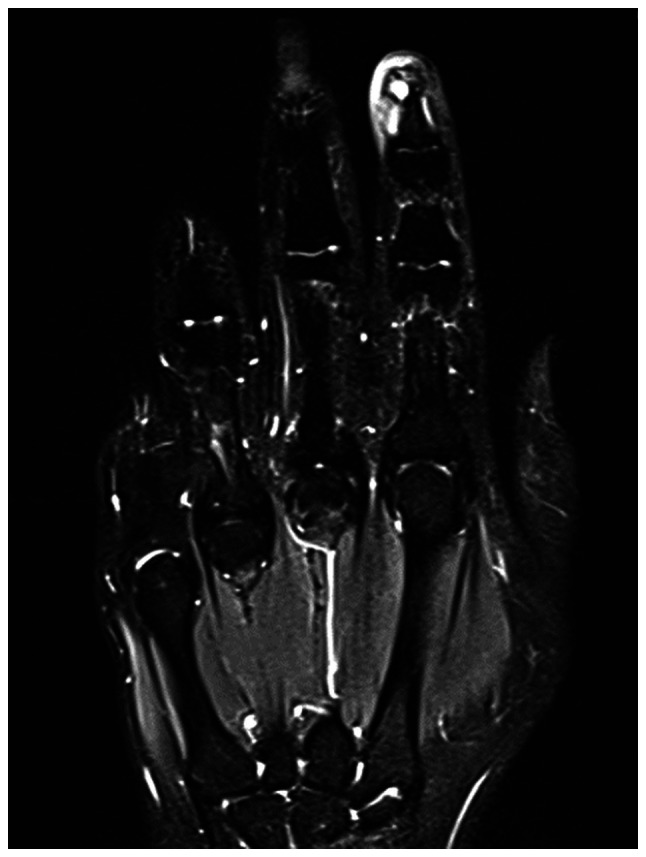
Coronal T2-weighted fat suppression showing high signal intensity.

**Figure 6.**
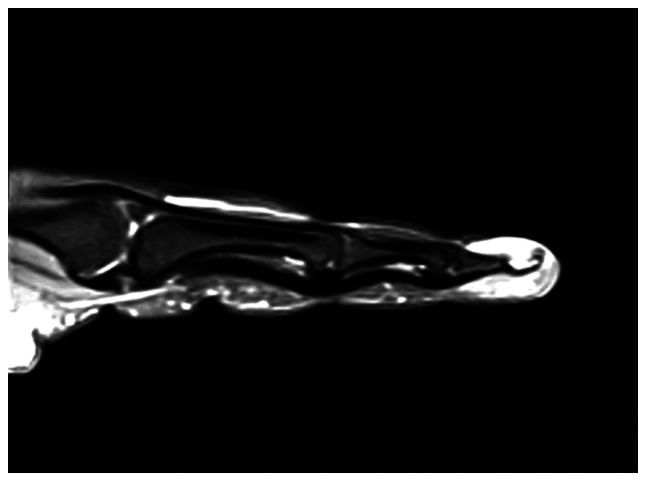
Sagittal proton density-weighted magnetic resonance imaging scan of the lesion.

Further evaluation with contrast-enhanced magnetic resonance imaging (MRI) was performed, including axial, sagittal and coronal projections on T1-weighted, fat-saturated-T2-weighted, proton density and post-contrast T1-weighted images. MRI revealed a well-defined, rounded soft tissue lesion seen dorsal to the distal phalanx of the right index finger, measuring 0.5×0.2×0.5 cm, demonstrating low signal intensity on the T1-weighted images, high signal intensity on the T2-weighted images and intense homogenous enhancement on the post-contrast images (Fig. 3, Fig. 4, Fig. 5, Fig. 6). Moreover, surrounding subcutaneous tissue and bone marrow edema was observed, with underlying bone remodeling of the distal phalanx. No other lesions or fractures were observed. Considering the location and radiological features of the lesion; the initial impression suggested a glomus tumor.

**Figure 7.**
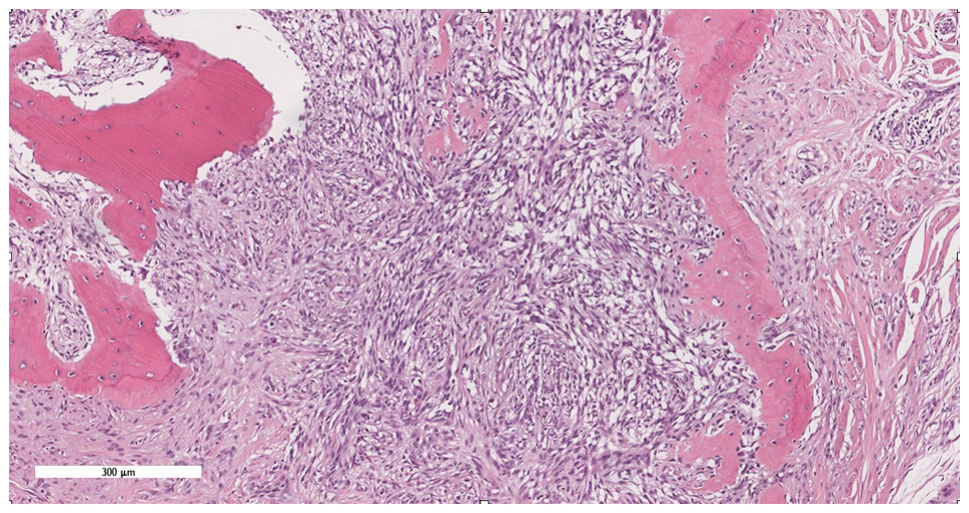
Section showing extensive infiltration by tumor cells into the underlying bone (hematoxylin and eosin stain; magnification, ×10).

**Figure 8.**
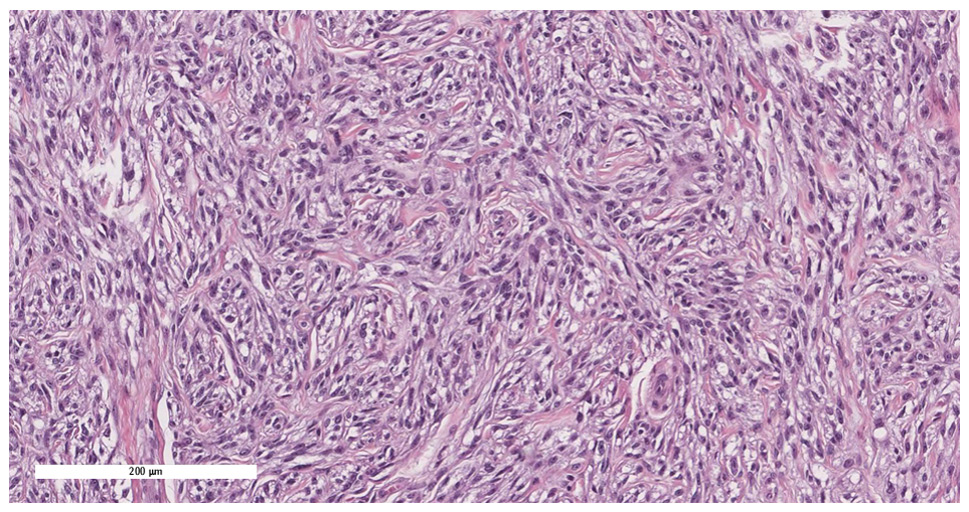
Tumor cells exhibited a spindle to epithelioid pattern, with eosinophilic to clear cytoplasm, vesicular nuclei and visible to prominent nucleoli (hematoxylin and eosin stain; magnification, ×20).

**Figure 9.**
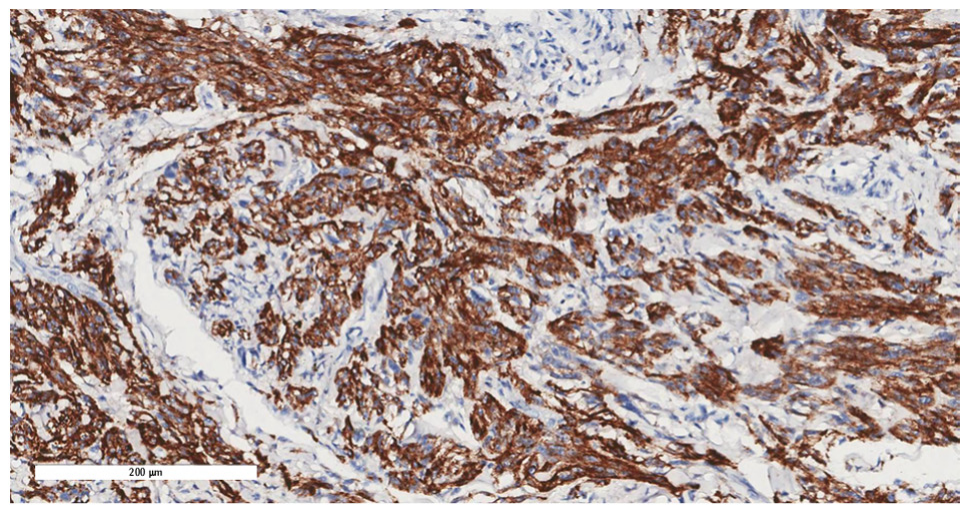
Tumor cells showing diffuse cytoplasmic staining for HMB-45 (immunohistochemical stain; magnification, ×20).

**Figure 10.**
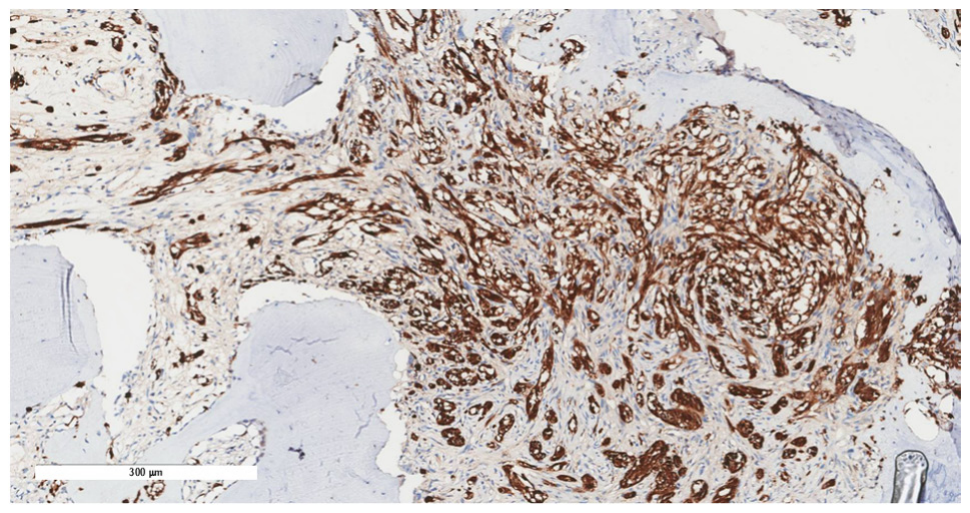
Tumor cells showing diffuse cytoplasmic and nuclear staining for S-100 protein (immunohistochemical stain; magnification, ×20).

Consequently, intralesional surgical excision of the lesion was performed using an ulnar-sided approach to the distal phalanx of the second finger with nail bed flap elevation; and the resected tissues were sent for histopathological assessment, as informed by previously published literature (10–12). Notably, the histopathological assessment revealed minute fragments of bone with fibrous tissue and entrapped clear cells suggestive of CCS (Fig. 7, Fig. 8, Fig. 9, Fig. 10). The tumor showed diffuse cytoplasmic staining for the immunohistochemical markers HMB-45 and S-100 protein, supporting the diagnosis of CCS. The results were subsequently sent to a specialized sarcoma pathologist in the oncology center King Faisal Specialist Hospital and Research Centre (Riyadh, Saudi Arabia), who concurred with the diagnosis.

Further local and systemic staging evaluation was performed and confirmed localized disease (stage II) using the American Joint Committee on Cancer staging system (13). Hence, the patient was informed and referred to a tertiary oncology center. A discussion at a multidisciplinary sarcoma tumor board meeting recommended local control with wide surgical resection by amputation through the middle phalanx, considering the potential contamination of the distal phalanx during the previous unplanned surgical resection. Neither local radiotherapy nor systemic chemotherapy was recommended by the tumor board, given the location and small size of the tumor and the absence of metastasis.

At the tertiary center, the proposed surgical treatment was explained to the patient and their parents, and informed consent was obtained. The patient underwent amputation through the middle phalanx of the right index finger, aiming to achieve local control with negative margins. During the procedure, meticulous dissection was performed to isolate digital neurovascular bundles, which were transected proximally while maintaining hemostasis. The flexor digitorum superficialis (FDS) tendon was preserved while flexor digitorum profundus tenotomy was performed at the base of the middle phalanx level, followed by tenodesis to the FDS tendon. An osteotomy was then performed of the middle phalanx at the proximal third level using a handheld oscillating saw. Complete amputation was achieved as planned, with negative margins. Primary closure of the amputation stump was conducted in layers, and a sterile dressing was applied.

**Figure 11.**
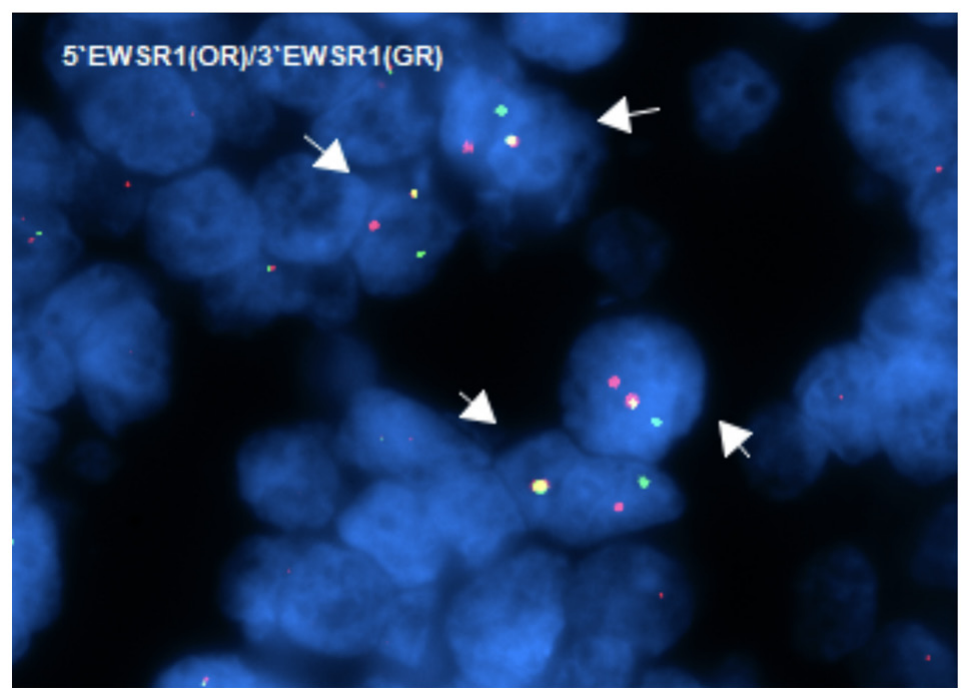
FISH for EWSR1 gene rearrangement in clear cell sarcoma. Dual-color break-apart probe demonstrated separation of the 5′ (orange) and 3′ (green) EWSR1 signals (arrows), indicating a rearrangement of the EWSR1 locus at 22q12 (magnification, ×1,000, oil immersion). FISH, fluorescence *in situ* hybridization.

The received specimen was preserved in formalin and comprised a finger segment measuring 4.5×2.0×1.5 cm. Examination of the segment revealed an ulcerated, ill-defined mass at the nail tip, measuring 0.7×0.4×0.4 cm, grossly abutting the underlying bone. The ulcerated mass was situated 2.5 cm from the resection margin. The diagnosis confirmed the presence of CCS in the distal phalanx of the right index finger. The tumor, measuring 0.7 cm at its greatest dimension, extended from the subungual soft tissue to the underlying bone and demonstrated perineural invasion. All resection margins, including the bone margin, were free of tumor, indicating successful surgical removal without residual malignant tissue. The specimen was sent for cytogenetic study (fluorescence *in situ* hybridization) (14); the results were positive for EWSR1 (22q12) gene rearrangement confirming the diagnosis of CCS (3) (Fig. 11).

**Figure 12.**
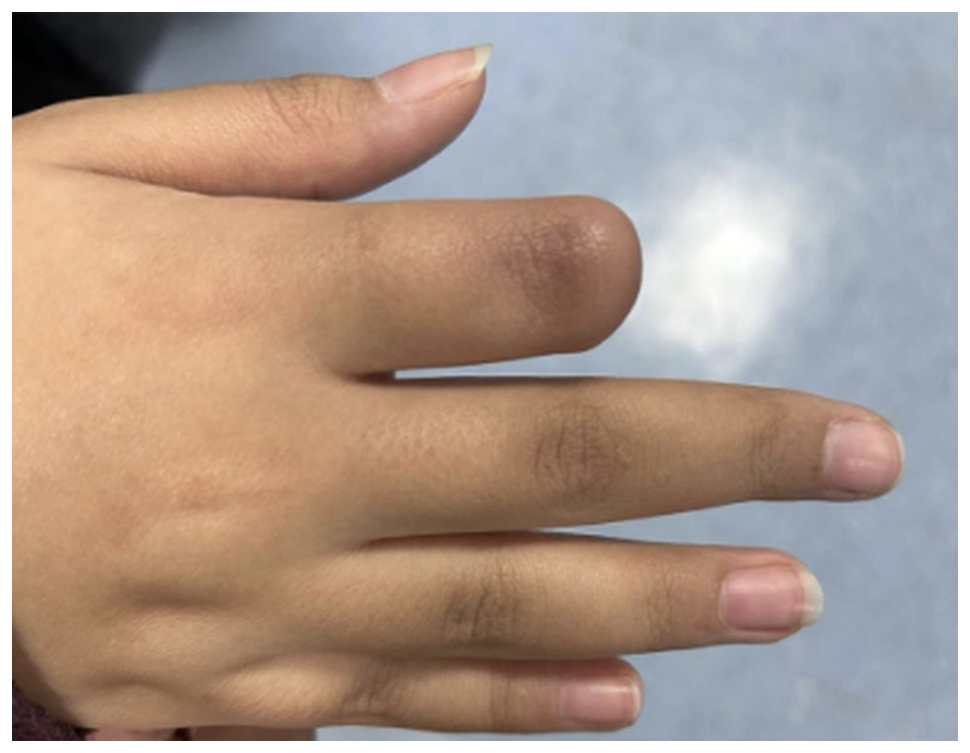
Right hand following amputation of the index finger.

**Figure 13.**
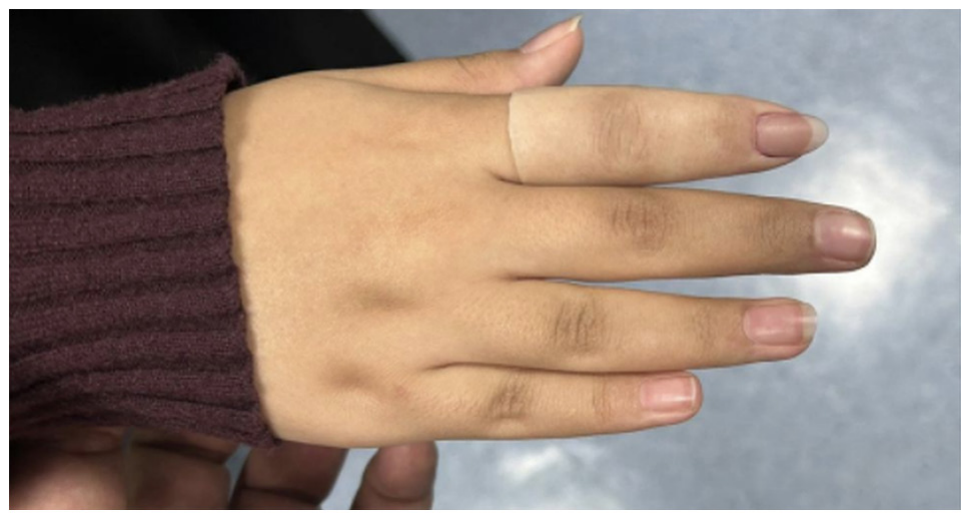
Right hand of the patient with a well-fitted prosthetic finger.

The patient has since had routine surveillance follow-up visits at the orthopedic oncology clinic as per sarcoma protocol, with the most recent follow-up being 24 months postoperatively. The patient initially experienced phantom and neuropathic pain at the amputation stump, which was subsequently resolved with multimodal analgesia. They also underwent physiotherapy and occupational therapy during the postoperative course. The hand function of the patient has been preserved, and they can perform daily activities after being fitted with a silicone prosthetic finger (Figs. 12 and 13). They have high functional scores of 86.6 out of 100 for the Musculoskeletal Tumor Society scoring system for the upper extremity, and 1.7 out of 100 for the Disabilities of Arm, Shoulder, and Hand score (15,16), suggesting minimal disability.

## Discussion

CCS was initially described as a ‘malignant melanoma of soft parts’ due to histological similarities; however, CCS can be challenging to diagnose as it resembles more common benign lesions, leading to inappropriate management or delayed referral to specialized facilities (17,18). Plain radiographs and MRI are the standard-of-care initial investigations for assessing CCS (19,20), and biopsy is the gold standard to confirm a histopathological diagnosis (21). In gross appearance, CCS resembles a well-defined solid tumor that is matte gray in color, and typically infiltrates tendons and aponeuroses. Microscopically, findings are small compact nests with uniform neoplastic cells divided into variably sized clusters by fibrous septa along the tendons and aponeuroses (1,22). Most CCSs are associated with a t(12;22)(q13-14;q12) translocation, which corresponds to the EWSR1 (22q12) gene rearrangement (23,24), which is the same translocation identified in the current case. Treatment of CCS involves local control with free margin excision of the tumor as soon as the diagnosis is established, with no recorded benefits from chemotherapy and radiotherapy (18,25). In addition, some studies have indicated that chemotherapy or radiotherapy has minimal effect at stopping CCS recurrence, particularly when there is an inadequate margin of excision (26–28). The presence of necrosis, metastasis and local recurrence, as well as a tumor size >5 cm, are poor prognostic factors (18,25). The overall estimated 5- and 10-year survival rates are ~50 and 38%, respectively, with the lung being the most common site of metastasis (5).

For the present case report, a literature review of all reported cases of CCS involving the fingers was conducted through searches of the PubMed (https://pubmed.ncbi.nlm.nih.gov/), Google Scholar (https://scholar.google.com/) and Web of Science (https://www.webofscience.com) databases. The search terms included ‘clear cell sarcoma’, ‘CCS’, ‘hand’ and ‘finger’. Each publication was reviewed, and six cases of CCS of the finger were identified (Tables I and II) (29–34). Percentages were calculated for all six patients showing the distribution of clinical and pathological features; a dash in the table indicates the absence of the feature, and missing information from the Maiorana *et al* (33) case was classified as ‘unknown’ due to the original full report not being available. Patients were aged between 13 and 59 years (median age: 42 years), with three male patients (50%) and three female patients (50%). Cases originated from the USA (two patients), Japan (one patient), Turkey (one patient), Iran (one patient) and Italy (one patient). Clinical presentation involved having a painful mass or nodule (four patients, 67%), while one patient (17%) presented with a painless mass and one case (17%) was unknown due to incomplete reporting (33). A history of trauma was documented in three patients (50%), absent in two (33%) and unknown in one (17%). Tumor size was available in five patients (83%) and ranged from 1.0×1.0×2.0 cm to 6×8 cm; the size was not retrievable in one patient (17%) (33). Cytogenetic confirmation of the EWSR1 translocation was not reported in any of the six cases; it was demonstrated only in the patient described in the present study. Metastatic spread was documented in four patients (67%) absent in one patient (17%) and unknown in one case (17%). In one case, axillary node, lung and bone metastases were reported after 6 years, with a survival of 6.5 years (29), and in another case, axillary node and lung metastases within 1.5 years were described (30). Lung and brain metastases within 9 and 14 months of diagnosis, respectively, were observed in a further case (31). Pulmonary metastases were also reported in another report, and the patient died after a short period (32). One of the earliest reports could not be fully retrieved, which accounts for missing clinical details in the analysis (33). Overall, the available literature highlights the rarity of CCS in the finger, its wide age distribution, and the potential for both early and delayed metastatic spread despite initial local control (29–34).

Compared with existing case reports, the present case shares histopathological and molecular findings, including the presence of diffuse cytoplasmic staining for immunohistochemical markers HMB-45 and S-100 protein (10,11), which have been reported (32,34). However, unlike cases presenting with poor prognostic factors (29,32), the patient described in the current report presented with a well-circumscribed tumor <5 cm and no evidence of distant metastasis, suggesting a favorable prognosis. Consistent with other cases emphasizing the challenge of early detection due to the resemblance of the tumor to other benign neoplasms (29,33), the present case study shows that prompt recognition is key to achieving optimal outcomes.

It is essential for clinicians to maintain a low threshold for considering CCS, given its slow-growing yet aggressive nature. Early detection and intervention are still important, as these tumors often remain small for years before rapidly metastasizing. The initial misdiagnosis as a glomus tumor highlights the risk of unplanned excisions in digital lesions, which can compromise margins in the amputation and general outcomes, highlighting the need for early biopsy and molecular confirmation in such presentations. The patient described in the current study is currently being followed up once every 3 months, and is now 2 years post-amputation, has had no recurrence and is doing well.

Ultimately, a high index of suspicion and timely diagnosis can improve outcomes, but the inherent biology of CCS poses notable challenges, even with appropriate management. Given the small number of reported cases, it is difficult to establish definitive treatment protocols for CCS, particularly considering the limited number of pediatric cases. However, the fact that metastasis often occurs long after the appearance of the primary lesion suggests that vigilant long-term monitoring is necessary. Additionally, the present findings support the notion that amputation remains an effective treatment even in the case of recurrence, emphasizing the potential value of early, aggressive surgical intervention. These insights may help to guide future treatment decisions, especially in the absence of dissemination, and could contribute to diagnostic guidelines for CCS in pediatric populations.

In conclusion, to the best of our knowledge, only six cases of CCS presenting in the finger have been reported in the literature to date. The current case represents the second pediatric case reported and the only one with confirmed EWSR1 translocation, further contributing to the limited literature. This highlights the rarity of CCS presentation in the hand, and emphasizes the importance of maintaining a high index of suspicion to identify, accurately diagnose, avoid mistreatment and prevent delays in referral to specialized centers. In addition, including CCS in the differential diagnosis of atypical digital lesions is needed. Multidisciplinary management in a specialized oncology center is crucial to optimize oncological and functional outcomes. Finally, future research should focus on exploring genetic markers and prognostic factors, and developing optimal management strategies for CCS, especially in rare presentations, to improve diagnostic accuracy and treatment outcomes.

## Figures and Tables

**Table I.** Demographic and clinical features of reported clear cell sarcoma cases of the finger.

First author, year	No. of patients	Year	Age	Sex	Country	Clinical presentation	History of trauma	(Refs.)
Ozuguz, 2014	1	2014	58	Female	Turkey	Painful mass	No	(32)
Tyler, 1980	1	1980	13	Female	USA	Painful firm mass	Yes	(29)
Tavakoli, 2011	1	2011	56	Male	Iran	Painless mass	Yes	(34)
Raynor, 1979	1	1979	59	Male	USA	Painful nodule	Yes	(30)
Maiorana, 1979	1	1990	28	Female	Italy	N/A	N/A	(33)
Ikeda, 1996	1	1996	17	Male	Japan	Painful mass	No	(31)

N/A, not available.

**Table II.** Tumor features and outcomes of reported clear cell sarcoma cases of the finger.

First author, year	Tumor size, cm	Translocation	Metastasis	Interval between lesion appearance and metastasis, years	Recurrence	Survival	Remarks	(Refs.)
Ozuguz, 2014	6×8	-	Lung	-	-	-	Patient died after a short period	(32)
Tyler, 1980	1×1×2	-	Axillary nodes, lungs and bone	6	-	6.5 years	-	(29)
Tavakoli, 2011	3×4×4	-	-	-	-	-	-	(34)
Raynor, 1979	2.2	-	Axillary nodes, lungs	1.5	-	-	-	(30)
Maiorana, 1979	N/A	N/A	N/A	N/A	N/A	N/A	-	(33)
Ikeda, 1996	1.8×1	-	Lung, brain	9-14 months	-	-	-	(31)

N/A, not available (full report unavailable); -, not reported.

## Data Availability

The data generated in the present study are included in the figures and/or tables of this article.
